# Comparative analysis of autonomic effects of ketamine-xylazine in normotensive and hypertensive rats

**DOI:** 10.1590/1414-431X2025e14598

**Published:** 2025-08-29

**Authors:** P.S. Santos, J.L. Pereira, S.S.P. Araújo, J.A. da Silva, A.F.M. da Silva, J.G.V. de Assunção, R.G. Silva, A.A. de Oliveira, F.V.S. Nunes, M.S. Noleto, A.P. de Oliveira, R.N. Soriano, L.G.S. Branco, H.C. Salgado, J.P.J. Sabino

**Affiliations:** 1 Departamento de Biofísica e Fisiologia, Universidade Federal do Piauí, Teresina, PI, Brasil; 2 Departamento de Ciências Básicas da Vida, Universidade Federal de Juiz de Fora, Governador Valadares, MG, Brasil; 3 Departamento de Biologia Básica e Oral, Faculdade de Odontologia de Ribeirão Preto, Universidade de São Paulo, Ribeirão Preto, SP, Brasil; 4 Departamento de Fisiologia, Faculdade de Medicina de Ribeirão Preto, Universidade de São Paulo, Ribeirão Preto, SP, Brasil

**Keywords:** Autonomic nervous system, Anesthetics, Cardiovascular system, SHR

## Abstract

This study aimed to evaluate the effects of the ketamine-xylazine (KX) anesthetic mixture on autonomic and cardiovascular functions in normotensive rats (Wistar) and spontaneously hypertensive rats (SHR), using both spectral and symbolic analyses. Male Wistar (n=22) and SHR (n=28) rats were intramuscularly anesthetized with KX, and their femoral artery and vein were cannulated for pulsatile arterial pressure recording and drug administration. Autonomic function was assessed 24 and 48 h post-surgery through spectral and symbolic analyses of heart rate (HR) and systolic arterial pressure (SAP) variability. KX anesthesia significantly decreased mean arterial pressure (MAP) 24 h post-surgery in both Wistar and SHR rats. Spectral analysis revealed increased sympathetic modulation in the vascular bed of SHR 48 h post-surgery. In Wistar rats, there was a significant reduction in parasympathetic modulation at 48 h, as indicated by root mean square of successive RR interval differences (RMSSD) and high frequency (HF) (nu) indices. Symbolic analysis, however, detected no significant changes in autonomic modulation. These data are consistent with the notion that KX anesthesia significantly impacts autonomic and cardiovascular functions, with differential effects observed between Wistar and SHR rats. Spectral analysis proved more effective than symbolic analysis in detecting these changes. These findings highlight the need for careful consideration of anesthetic effects in experimental research and suggest that optimizing anesthetic protocols could improve clinical outcomes by minimizing adverse autonomic impact.

## Introduction

Numerous experimental models are used in basic research, including rodents such as rats and mice, which are commonly used in studies evaluating cardiovascular (1) and autonomic (2) functions under normal (Wistar) and pathological conditions (spontaneously hypertensive rat (SHR) model) (3,4). The selection of an anesthetic is an important step in experimental studies involving surgical procedures, as it influences the survival of the animals and can interfere with the results; it is well known that anesthetics can alter various physiological parameters, such as arterial pressure (AP) and heart rate (HR) (5). In this regard, there are a variety of anesthetics utilized in experimental research, with the ketamine-xylazine (KX) anesthetic mixture being widely used (6).

Ketamine is a dissociative and non-competitive general anesthetic that alters central nervous system (CNS) functions and stimulates the sympathetic nervous system (SNS). Its main effects include analgesia, amnesia, and immobility. However, since it does not promote adequate skeletal muscle relaxation, it is often used in combination with another anesthetic, primarily xylazine, an α2-adrenergic agonist. Xylazine causes CNS depression and has sedative, analgesic, and muscle relaxant effects (7,8).

As mentioned, general anesthetics alter the CNS, influencing the SNS and parasympathetic nervous system (PNS) balance (9,10). The autonomic nervous system (ANS) modulates the cardiovascular system by releasing norepinephrine and acetylcholine in the heart, leading to changes in HR and force of contraction of myocardial fibers, and by releasing mainly norepinephrine in systemic resistance blood vessels, which results in increased peripheral vascular resistance (11). Therefore, general anesthetics affect ANS interpretation and cardiovascular outcomes (10,12).

Regarding the tools for assessing ANS control in the cardiac muscle, spectral analysis has been widely used alongside non-linear methods for HR variability (HRV) analysis (13-15). Similarly, the analysis of AP variability (APV) provides information about the autonomic modulation of vessels, particularly reflecting the sympathetic action on resistance blood vessels (16). In this context, the cardiovascular variability analysis has become a powerful non-invasive tool to quantify autonomic modulation under different physiological conditions. It highlights the ability to identify improvements in autonomic modulation, such as after a physical activity protocol (17) or pharmacological treatment (18), as well as to detect deterioration in autonomic modulation with prevalence in sympathetic activity, as observed in cardiovascular diseases (19). Additionally, there is a growing body of evidence that suggests that HRV and APV can be used as predictors of mortality and aging rates (20,21).

Although cardiovascular variability is a low-cost and easily obtained measure of autonomic function, it is essential to implement procedures to optimize analysis quality in order to generate data that accurately reflect reality. Therefore, understanding how an anesthetic affects cardiovascular autonomic analysis can help determine which analysis tools are most efficient in assessing autonomic modulation under a given experimental condition.

Against this background, the present study aimed to evaluate whether spectral and/or symbolic analyses (non-linear methods) can detect any influence of the anesthetic mixture (KX) on the autonomic modulation of HR and AP in Wistar and SHR rats 24 and 48 h post-surgery.

## Material and Methods

### Animals and experimental groups

Male Wistar (n=22) and SHR (n=28) rats aged 16±4 weeks and weighing between 280 and 340 grams were obtained from the Animal Facility of the Department of Biophysics and Physiology [Federal University of Piauí (UFPI)] and housed in plastic cages under controlled temperature conditions (22±2°C), 12 h light/dark cycle, with free access to water and food. Experiments were conducted at the Laboratory for the Study of Reflex Control of Arterial Pressure and Pulmonary Ventilation (LEPAVE; Department of Biophysics and Physiology, UFPI). All experimental procedures were conducted in accordance with the guidelines of the National Council for Animal Experimentation Control (CONCEA) and approved by the Ethics Committee for Animal Use of UFPI (CEUA/UFPI/563/19). The animals were grouped into 4 experimental groups: 1) Wistar 24 h (n=8), 2) Wistar 48 h (n=14), 3) SHR 24 h (n=11), and 4) SHR 48 h (n=17).

### Surgical procedure

The animals were anesthetized with ketamine (33.33 mg/kg) and xylazine (13.3 mg/kg), and the femoral artery and vein were cannulated for recording pulsatile arterial pressure (PAP) and drug administration, respectively. The femoral artery and vein cannulation surgery consisted of a small surgical incision in the inguinal region, followed by isolation of the femoral artery and vein after locating the femoral neurovascular bundle. Subsequently, polyethylene (PE) cannulas filled with saline solution (0.9% NaCl) were inserted into the blood vessels. The cannulas were made with PE-10 segments (Intramedic, Becton Dickinson and Company, USA), venous cannula (3 cm), and arterial cannula (5 cm), connected to 18-cm segments of PE-50 (Intramedic, Becton Dickinson and Company). After inserting the PE-10 segment into the femoral artery and vein, the free ends of the cannulas (PE-50) were subcutaneously conducted to the dorsal area of the animals using a trocar, exteriorized in the interscapular region, and secured with suture thread. After surgery, the animals received 100 mg/kg of ketoprofen *iv*. Subsequently, the rats were placed in individual cages for surgical recovery with free access to water and food.

### Pulsatile arterial pressure (PAP) recording

To record PAP (24 and 48 h after surgical recovery), the femoral artery cannula was connected to a pressure transducer (MLT0699, ADInstruments^®^, Australia) coupled to a signal amplifier (GP Amp, ADInstruments^®^) and a data acquisition system (Power Lab 26 T, ADInstruments^®^). The software used for processing the PAP recording was LabChart 7.0 (ADInstruments^®^). Monitoring included an acclimatization period (60 min), followed by baseline PAP recording (30 min) at 2 kHz sampling frequency. Measures were taken to reduce the animals' stress during monitoring, such as placing them in a quiet, isolated environment with minimal human movement, and each animal was placed in a separate box. The experiments were conducted between 8:00 a.m. and 3:00 p.m.

From the PAP recording, mean AP (MAP), systolic AP (SAP), and HR values were obtained. At the end of the experimental protocol, all animals were euthanized with a supraphysiological dose of sodium thiopental (100 mg/kg, intravenously), following the guidelines established by Resolution No. 1,000, May 11, 2012, of the Federal Council of Veterinary Medicine (CFMV, Brazil).

### Analysis of SAP and HRV

Spectral analysis is one of the linear methods for analyzing HRV, which can be performed in the time and frequency domains. The time domain determines the variation in the duration of intervals between QRS complexes and presents its results via statistical indices, such as the RMSSD (root mean square of successive RR interval differences), an index of parasympathetic modulation. In the frequency domain, the analysis of the cardiac interval recording includes the following indices: HF (high frequency), an indicator of vagus nerve activity on the heart, LF (low frequency), representing the combined modulation of the parasympathetic and sympathetic components on the heart, with a predominance of the sympathetic component, VLF (very low frequency), and ULF (ultra-low frequency), which are rarely used due to their less well elucidated physiological explanations (22,23).

AP is a physiological parameter characterized by continuous fluctuations all the time. The size and patterns of these variations characterize APV. APV can be assessed by measuring the dispersion of means, for example, standard deviation, and by estimates that also consider a series of measurements over time. Among the most current methods for evaluating APV, the spectral analysis technique is the most commonly used (24).

Nonlinear methods are also used to assess HRV, highlighting symbolic analysis, which involves converting a series of HR interval variability into a sequence of symbols, transformed into patterns, reduced, and grouped into a small number of families, and subsequently, the occurrence rates of these families are evaluated. In this way, R-R intervals are classified into 6 levels (0 to 5) and grouped into sequences of 3 symbols, classified into 4 families: 0V%, indicating sympathetic modulation; 1V%, indicating no association with sympathetic and parasympathetic modulation; 2LV% and 2UV%, both associated with parasympathetic modulation (25-28).

Analysis of SAP variability (SAPV) and HRV in the time and frequency domains was carried out using the CardioSeries^®^ 2.7 software (available at http://www.danielpenteado.com/cardioseries). Symbolic analysis, a non-linear method for evaluating HRV, was also performed using the computer program mentioned above. In LabChart 7.0 (ADInstruments^®^), stable segments were selected from the PAP recordings of approximately 10-min duration in order to extract time series with SAP (mmHg) and pulse interval (PI) (ms) values. The segments with the least interference from the external environment on the animal were selected for analysis. The file containing these values was then converted into a compatible format for analysis in CardioSeries^®^ 2.7.

In the time domain, SAPV was evaluated using the mean, standard deviation (SD), and variance, while HRV was analyzed using the mean, SD, variance, and RMSSD. In the frequency domain, SAPV and HRV were calculated by spectral analysis using the Fast Fourier Transform method. Based on the data obtained by software processing, the oscillatory components were integrated into LF (low frequency: 0.20 to 0.75 Hz) and HF (high frequency: >0.75 to 3.00 Hz) bands. The LF/HF ratio was also calculated to assess sympathovagal balance. LF and HF values were expressed in normalized units (nu) for PI and SAP, and LF values were expressed in absolute units (mmHg^2^). In the symbolic analysis, the PI segments were divided into 6 levels (0 to 5), sequenced, and grouped into 3 families: 0V, 1V, and 2V (obtained by adding the 2LV and 2UV families, according to the methodology by Nascimento et al. (28), expressed as percentage (%).

### Statistical analysis

Statistical analysis was performed using Sigma Plot^®^ 11.0 (Systat Software, Inc., Germany) and Graph Pad Prism^®^ 8.0 (Graph Pad Software, Inc., USA), and the results are reported as means±SEM. The Shapiro-Wilk test was used to assess the normality of data distribution.

Group comparisons were performed using Student's *t*-test for normally distributed data and the Mann-Whitney test for non-normally distributed data. Differences between groups were considered to be statistically significant at P<0.05.

## Results

**Figure 1:**
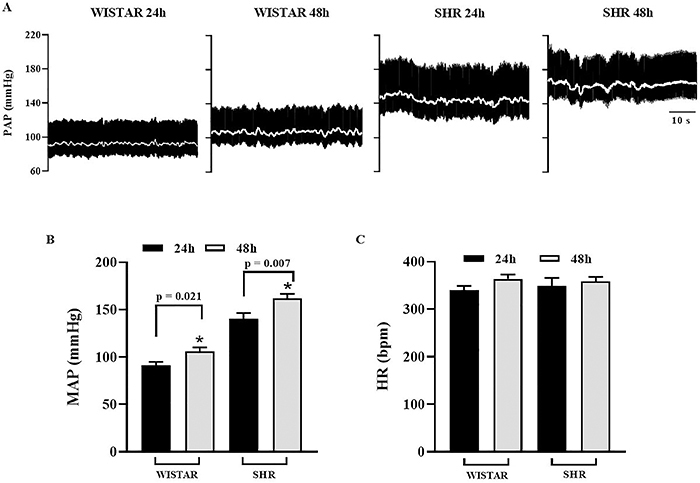
**A**, Representative trace of pulsatile arterial pressure (PAP, mmHg) and mean arterial pressure (MAP, white lines). **B**, Means±SEM of MAP and **C**, means±SEM of heart rate (HR) of Wistar and spontaneously hypertensive rat (SHR) groups at 24 and 48 h post-surgery. *P<0.05, Student's *t*-test.

Figure 1 shows a representative recording, equivalent to one minute of monitoring, comparing the PAP (black line) and MAP (white line) among different groups. Lower MAP values were observed in the 24-h Wistar and SHR groups compared to the 48-h groups. However, regarding HR, no statistical difference was observed among groups, although there was a slight increase in HR in rats monitored for 48 h.

**Figure 2:**
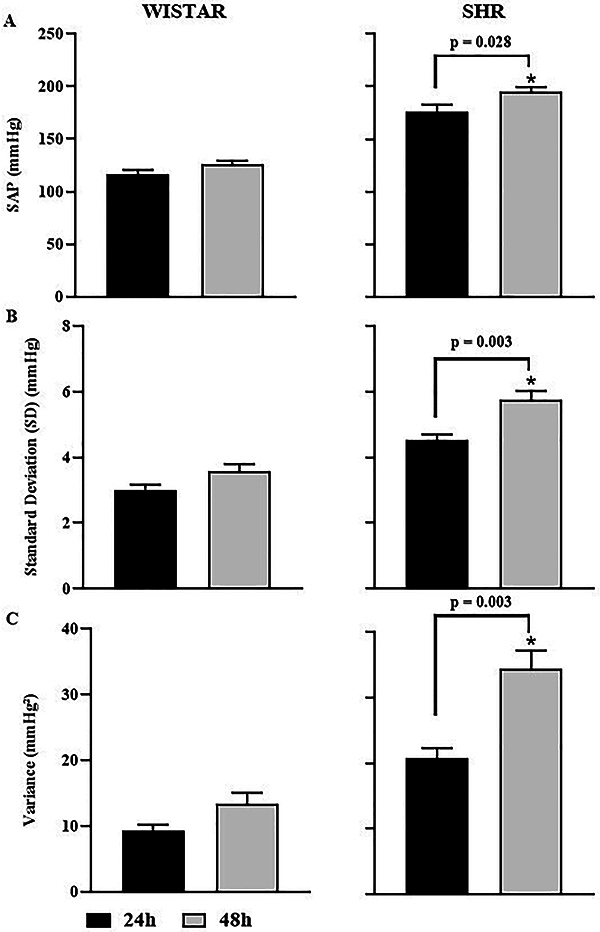
Indices of systolic arterial pressure (SAP) variability (SAPV) in the time domain of Wistar and spontaneously hypertensive rat (SHR) groups at 24 and 48 h of SAP (**A**), standard deviation (**B**), and variance (**C**). Data are reported as means±SEM. *P<0.05, Student's *t*-test.

In the analysis of SAP in the time domain, KX anesthesia attenuated SAP in SHR rats at 24 h compared to the 48-h group (Figure 2). On the other hand, regarding Wistar rats, KX did not alter SAP after 24 and 48 h post-surgery. There was a significant increase in standard deviation and variance in the SHR 48-h group compared to the 24-h group. However, in Wistar rats, no statistical difference was observed regarding these variables.

**Figure 3:**
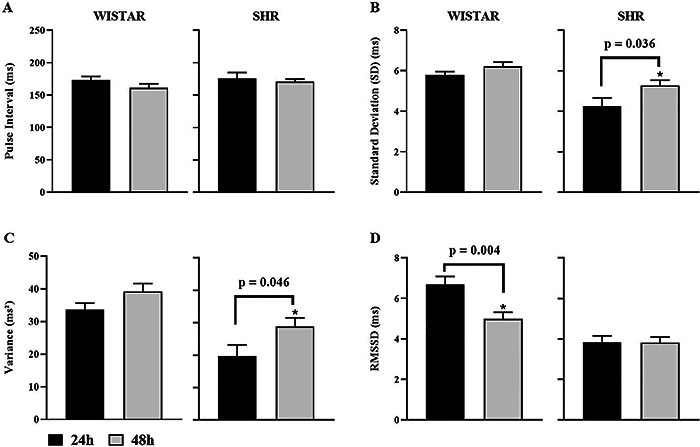
Indices of pulse interval (PI) variability in the time domain of Wistar and spontaneously hypertensive rat (SHR) groups at 24 and 48 h. PI (**A**), standard deviation (**B**), variance (**C**), and root mean square of successive RR interval differences (RMSSD) (**D**). Data are reported as means±SEM. *P<0.05, Student's *t*-test.

Regarding HRV in the time domain (Figure 3), in Wistar rats, the RMSSD (ms), corresponding to parasympathetic modulation of the heart, was greater at 24 h than at 48 h, while in the SHR, no change was observed in this parameter. In addition, there were no significant changes in PI in either group, but there was a significant increase in standard deviation and variance in the SHR 48-h group compared to the SHR 24-h group.

**Figure 4:**
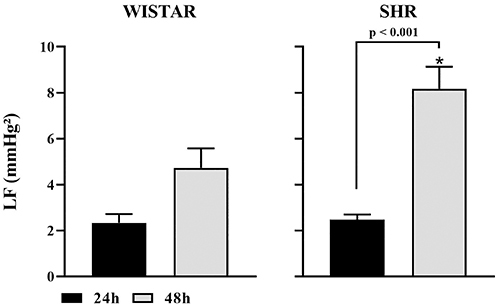
Low frequency (LF) oscillatory component (mmHg^2^) in the frequency domain of systolic arterial pressure (SAP) spectrum of Wistar and spontaneously hypertensive rat (SHR) groups at 24 and 48 h. Data are reported as means±SEM. *P<0.05, Student's *t*-test.

In the frequency domain of the SAP spectrum, a significant increase in the LF oscillatory component (mmHg^2^), corresponding to sympathetic modulation, was observed in SHR rats monitored 48 h after surgical recovery. However, in Wistar rats, although the LF component was higher at 48 h, as shown in Figure 4, this increase was close to statistical significance (P<0.053). Thus, Wistar rats monitored for 48 h post-surgery showed a trend towards increased sympathetic modulation on SAP, represented by LF.

**Figure 5:**
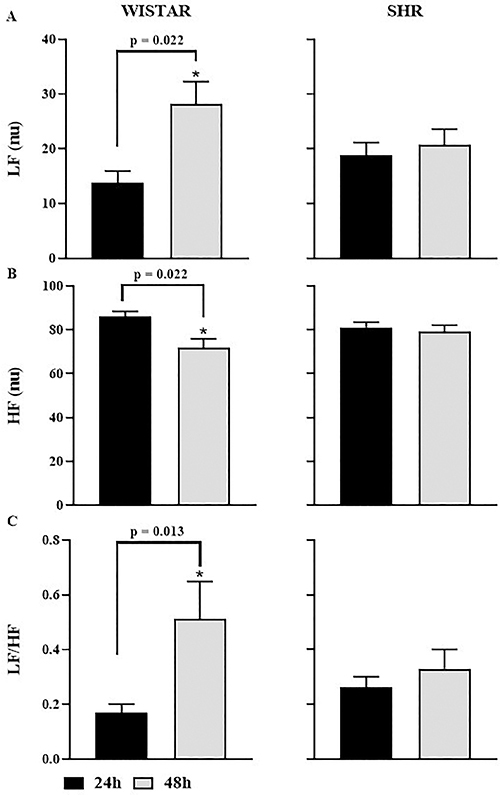
Pulse interval (PI) variability indices in the frequency domain of Wistar and spontaneously hypertensive rat (SHR) groups at 24 and 48 h. Low frequency (LF) (**A**), high frequency (HF) (**B**), and sympathovagal balance (LF/HF) (**C**). Data are reported as means±SEM. *P<0.05, Student's *t*-test.

Figure 5 illustrates the effect of KX anesthesia on the autonomic response of the PI in the frequency domain. In Wistar rats, we observed an increase in sympathetic modulation corresponding to the LF component and a decrease in HF, which indicates parasympathetic modulation, leading to an increase in sympathetic-vagal balance (LF/HF) in the 48-h group compared to the 24-h group. However, no difference was observed in SHR, although graphically, a slight increase in the LF band and the LF/HF ratio was seen in the 48-h group.

**Figure 6:**
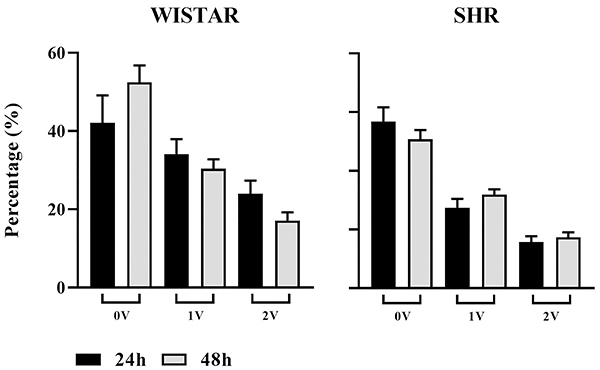
Symbolic analysis of pulse interval (PI) from Wistar and spontaneously hypertensive rat (SHR) groups at 24 and 48 h expressed as percentile of sympathetic (0V%) and parasympathetic modulation (2V%) and 1V%. Data are reported as means±SEM.

The autonomic assessment by symbolic analysis of the PI (Figure 6) did not detect changes in the 0V% (indicative of sympathetic modulation) and 2V% (indicative of parasympathetic modulation) parameters after the administration of the KX anesthetics in both Wistar and SHR rats, regardless of the surgical recovery period.

## Discussion

The anesthesia of experimental animals enables the execution of invasive surgical techniques that allow researchers to perform experimental protocols that are unfeasible in clinical studies. Thus, information regarding anesthetic side effects or limitations of an experimental approach facilitates the interpretation of research results and assists in determining the optimal timing for conducting an experimental protocol. The main objective of the present study was to investigate whether the effects of the KX anesthetic mixture on the ANS could be detected by spectral and symbolic analyses in normotensive and hypertensive rats, in an attempt to provide support for studies of autonomic control on cardiovascular variability, which could minimize the potential adverse effects of anesthetics on the outcomes. Alongside the autonomic analysis, we also evaluated the adverse effects of KX on AP and HR in Wistar rats and SHR 24 and 48 h post-surgery.

**Figure 7:**
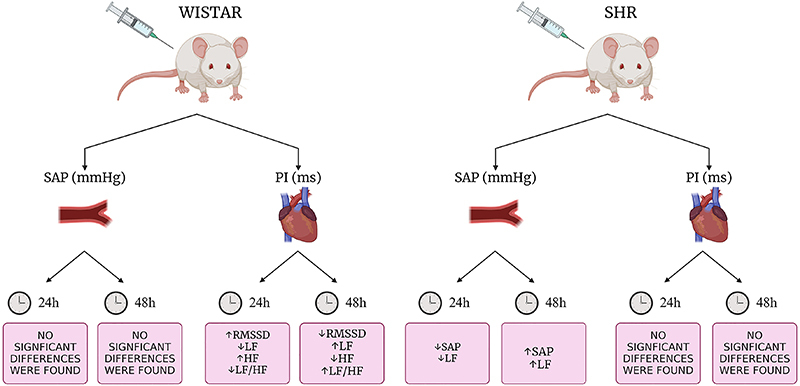
Schematic image of the findings showing the effect of the anesthetic mixture ketamine-xylazine on heart rate and systolic arterial pressure variability in Wistar rats and spontaneously hypertensive rats (SHR) monitored for 24 and 48 h. PI: pulse interval; HF: high frequency; LF: low frequency; RMSSD: root mean square of successive RR interval differences; SAP: systolic arterial pressure. Created with BioRender.com.

The results of the present study indicated that the KX anesthesia appears to significantly alter the autonomic nervous system and cardiovascular system 24 h after administration, causing a decrease in MAP in both strains compared to the 48-h group. This result is supported by the analysis of sympathetic modulation on the vascular bed, which showed an increase in the LF power band of SAP 48 h after cannulation. Additionally, 48 h post-surgery, only Wistar rats showed attenuation of RMSSD and HF (nu) indices, indicating a reduction in parasympathetic modulation on PI, along with an increase in sympathetic modulation due to the increase of LF (nu) and LF/HF parameters. Meanwhile, SHR showed improvements in variance and standard deviation indices, suggesting an improvement in total variability. The main findings of the present study are illustrated in Figure 7.

We also evaluated HR, and the findings showed that the administration of KX did not cause changes in this parameter (24- and 48-h time points). Some studies where the KX anesthetic mixture was administered intravenously showed that acute administration of the anesthetic caused a decrease in HR and MAP in Wistar rats (29,30). Additionally, the hypotension observed in the first 24 h of the present study may have started immediately after anesthetic administration, as previous studies demonstrated a drop in MAP and HR immediately after KX injection that persisted until the end of the experimental protocol (210 min) (31). Furthermore, studies demonstrate that ketamine clearance in rats can occur in 2.5 to 3 days with detection in plasma serum up to 10 days (32), while xylazine can be eliminated from the organism within 4 to 5 days (33).

As mentioned above, KX leads to changes in the autonomic modulation of HR and AP. In light of this, a recent study showed that intravenous administration of KX in mice led to sympathetic withdrawal, with a tendency towards vagal activation (34). Barboza (35) reported that intravenous administration of KX decreased HR and AP variability, with reductions in the LF and HF bands in the PI frequency domain spectrum. In the time domain, RMSSD values were also reduced by KX anesthesia, indicating a reduction in both sympathetic and parasympathetic modulation of the heart and blood vessels. Those findings partially corroborate the results of the present study (obtained 24 and 48 h after intramuscular administration) since a reduction in LF in the PI was observed in Wistar rats and a reduction in LF of AP was observed in SHR 24 h post-surgery compared to 48 h post-surgery. Moreover, the parasympathetic activity indices, RMSSD, and HF of PI in Wistar rats were higher 24 h after surgery. Therefore, in Wistar rats, KX had a greater impact on cardiac autonomic modulation, while in SHR, KX seemed to have a greater effect on sympathetic modulation of the vascular bed. However, these changes were only detected by spectral analysis.

Ketamine has a sympathomimetic effect, stimulating sympathetic activity and inhibiting the reuptake of catecholamines, leading to increased HR, AP, and cardiac output (CO) (36). However, the use of xylazine combined with ketamine can attenuate these effects on the cardiovascular system, possibly due to the inhibition of norepinephrine release through the stimulation of presynaptic adrenergic receptors by xylazine. This contributes to reducing the increase in SNS activity, inhibition of the baroreceptor reflex, and a decrease in vagal tone produced by ketamine (37). Thus, these physiological changes induced by the anesthetic mixture KX can explain the results obtained from AP.

Symbolic analysis is a non-linear method that has been widely used to evaluate the autonomic modulation of drugs on the cardiovascular system. However, few studies have highlighted the differences between linear and non-linear approaches by assessing the ANS in experimental models. Given this, Silva et al. (25) reported that symbolic analysis might be more effective in evaluating autonomic activity in rats than spectral analysis. Nevertheless, in the present study, spectral analysis was proven to be a more reliable method for assessing sympathetic and parasympathetic modulation of HR and AP.

While this study provides valuable insights into the effects of KX anesthesia on autonomic and cardiovascular functions in hypertensive and normotensive rats, several limitations should be acknowledged. First, the study's duration was relatively short, with observations limited to 24 and 48 h post-surgery. Longer-term studies are necessary to evaluate the sustained effects of these anesthetics on autonomic functions. Second, the study was conducted on animal models, which may not fully replicate the complexities of human physiology. Further research involving clinical trials or additional animal models is necessary to confirm the translatability of these findings to humans. Third, the study focused on specific parameters of HR and APV; other relevant physiological and molecular mechanisms underlying the observed effects were not explored in detail. Fourth, it is necessary to propose a new experimental approach capable of minimizing the side effects of KX anesthetic mixture, as in the present study, an increase in arterial pressure and alterations in autonomic variability were observed 48 h after anesthesia. However, we cannot assert that the effects of the anesthetic mixture were entirely exhausted after 48 h post-surgery. On the other hand, a current study suggests a probable anesthetic protocol capable of optimizing the anesthesia recovery. It was proposed that alternating between fasting and free access to water and food resulted in a shorter induction time for KX anesthesia in rats and a faster recovery compared to the non-fasting group (38).

Future research should include a broader range of biomarkers and mechanistic studies to elucidate the pathways involved. Finally, the exact dosing and timing of anesthesia administration may influence outcomes, and further studies are needed to optimize these variables for different experimental conditions. These limitations highlight the need for further research to validate and expand the findings presented in this study.

The present study demonstrated that the KX anesthetic mixture significantly alters autonomic and cardiovascular functions in both normotensive and hypertensive rats, with pronounced effects observed 24 h post-surgery. Specifically, KX anesthesia decreased MAP and altered HRV, particularly in Wistar rats, while spectral analysis indicated an increase in sympathetic activity in the vascular bed of SHR at 48 h. These findings suggest that KX anesthesia has a substantial and differential impact on autonomic modulation, emphasizing the need for careful consideration of anesthetic effects in experimental designs and data interpretation. Clinically, the present data stress the importance of optimizing anesthetic protocols to mitigate adverse autonomic effects, which could enhance patient outcomes in surgeries and other medical procedures. Moreover, the study's insights into the autonomic impacts of KX may inform the development of improved anesthesia management strategies, potentially reducing perioperative risks and improving cardiovascular health outcomes in both human and veterinary medicine. Future studies are needed to explore longer-term effects and different anesthetic combinations to fully elucidate the mechanisms and optimize clinical human and veterinary practices.

## References

[1] Jia T, Wang C, Han Z, Wang X, Ding M, Wang Q (2020). Experimental rodent models of cardiovascular diseases. Front Cardiovasc Med.

[2] Hélissen O, Kermorgant M, Déjean S, Mercadie A, Le Gonidec S, Zahreddine R (2023). Autonomic nervous system adaptation and circadian rhythm disturbances of the cardiovascular system in a ground-based murine model of spaceflight. Life (Basel).

[3] de Oliveira KBV, Severo JS, da Silva ACA, dos Santos BLB, Mendes PHM, Sabino JPJ (2023). P2X7 receptor antagonist improves gastrointestinal disorders in spontaneously hypertensive rats. Braz J Med Biol Res.

[4] Dos Santos RB, Oliveira LVC, Sena EP, de Sousa DP, Maia-Filho ALM, Soriano RN (2021). Acute autonomic effects of rose oxide on cardiovascular parameters of Wistar and spontaneously hypertensive rats. Life Sci.

[5] Cicero L, Fazzotta S, Palumbo VD, Cassata G, Lo Monte AI (2018). Anesthesia protocols in laboratory animals used for scientific purposes. Acta Biomed.

[6] Sancak T (2023). The effects of repeated doses of xylazine-ketamine and medetomidineketamine anesthesia on DNA damage in the liver and kidney. Acta Cir Bras.

[7] Wellington D, Mikaelian I, Singer L (2013). Comparison of ketamine-xylazine and ketamine-dexmedetomidine anesthesia and intraperitoneal tolerance in rats. J Am Assoc Lab Anim Sci.

[8] Levin-Arama M, Abraham L, Waner T, Harmelin A, Steinberg DM, Lahav T (2016). Subcutaneous compared with intraperitoneal ketamine-xylazine for anesthesia of mice. J Am Assoc Lab Anim Sci.

[9] Johnson JO (2019). Pharmacology and Physiology for Anesthesia.

[10] Savvina IA, Olegovna AP, Mikhailovna YZ, Aslanidis T & Nouris C (Orgs.) (2022). Physiology.

[11] Karim S, Chahal A, Khanji MY, Petersen SE, Somers VK (2023). Autonomic cardiovascular control in health and disease. Compr Physiol.

[12] Saha DC, Saha AC, Malik G, Astiz ME, Rackow EC (2007). Comparison of cardiovascular effects of tiletamine-zolazepam, pentobarbital, and ketamine-xylazine in male rats. J Am Assoc Lab Anim Sci.

[13] Maki KA, Goodyke MP, Rasmussen K, Bronas UG (2024). An integrative literature review of heart rate variability measures to determine autonomic nervous system responsiveness using pharmacological manipulation. J Cardiovasc Nurs.

[B14] De Angelis K, Santos MSB, Irigoyen MC (2004). Sistema nervoso autônomo e doença cardiovascular [in Portuguese]. Rev Soc Cardiol RGS.

[15] Ribeiro JP, Moraes-Filho RS (2005). Variabilidade da frequência cardíaca como instrumento de investigação do sistema nervoso autônomo. Rev Bras Hipertens.

[16] Yugar LBT, Yugar-Toledo JC, Dinamarco N, Sedenho-Prado LG, Moreno BVD, Rubio TA (2023). The role of heart rate variability (HRV) in different hypertensive syndromes. Diagnostics (Basel).

[17] da Silva JA, Araújo SSP, da Silva AFM, de Assunção JGV, Santos PS, Pereira-Júnior JL (2025). Chronic rose oxide and exercise synergistically modulate cardiovascular and autonomic functions in hypertensive rats. Pflugers Arch.

[18] Cavalcante GL, Brognara F, Oliveira LVC, Lataro RM, Durand MT, de Oliveira AP (2021). Benefits of pharmacological and electrical cholinergic stimulation in hypertension and heart failure. Acta Physiol (Oxf).

[19] Cavalcante GL, Ferreira FN, da Silva MTB, Soriano RN, Filho ALMM, Arcanjo DDR (2020). Acetylcholinesterase inhibition prevents alterations in cardiovascular autonomic control and gastric motility in L-NAME-induced hypertensive rats. Life Sci.

[20] Bencivenga L, Barreto PDS, Rolland Y, Hanon O, Vidal JS, Cestac P (2022). Blood pressure variability: a potential marker of aging. Ageing Res Rev.

[21] Jarczok MC, Weimer K, Braun C, Williams DP, Thayer JF, Gündel HO (2022). Heart rate variability in the prediction of mortality: A systematic review and meta-analysis of healthy and patient populations. Neurosci Biobehav Rev.

[22] Vanderlei LCM, Pastre CM, Hoshi RA, Carvalho TDD, Godoy MFD (2009). Basic notions of heart rate variability and its clinical applicability. Rev Bras Circ Cardiovasc.

[23] Figueiredo P, de Oliveira MIB, André SMS, do Nascimento DLA, Silva CSS, Rebouças GM (2013). Aplicabilidade Clínica da Variabilidade da Frequência Cardíaca. Rev Neuroci.

[24] Parati G, Stergiou GS, Dolan E, Bilo G (2018). Blood pressure variability: Clinical relevance and application. J Clin Hypertens (Greenwich).

[25] Silva LEV, Geraldini VR, de Oliveira BP, Silva CAA, Porta A, Fazan R (2017). Comparison between spectral analysis and symbolic dynamics for heart rate variability analysis in the rat. Sci Rep.

[B26] Cysarz D, Porta A, Montano N, Van-Leeuwen P, Kurths J, Wessel N (2013). Different approaches of symbolic dynamics to quantify heart rate complexity. Annu Int Conf IEEE Eng Med Biol Soc.

[B27] Porta A, Tobaldini E, Guzzetti S, Furlan R, Montano N, Gnecchi-Ruscone T (2007). Assessment of cardiac autonomic modulation during graded head-up tilt by symbolic analysis of heart rate variability. Am J Physiol Heart Circ Physiol.

[28] Nascimento L, Santos A, Lima A, Ritti-Dias R, Brasileiro-Santos M (2013). Comparação da análise simbólica da variabilidade da frequência cardíaca entre mulheres fisicamente ativas de meia-idade e idosas [in Portuguese]. Rev Bras Ativ Fis Saude.

[29] Shekarforoush S, Fatahi Z, Safari F (2016). The effects of pentobarbital, ketamine-pentobarbital and ketamine-xylazine anesthesia in a rat myocardial ischemic reperfusion injury model. Lab Anim.

[30] Sumitra M, Manikandan P, Rao KVK, Nayeem M, Manohar BM, Puvanakrishnan R (2004). Cardiorespiratory effects of diazepam-ketamine, xylazine-ketamine and thiopentone anesthesia in male Wistar rats: a comparative analysis. Life Sci.

[31] Picollo C, Serra AJ, Levy RF, Antonio EL, Santos LD, Tucci PJF (2012). Hemodynamic and thermoregulatory effects of xylazine-ketamine mixture persist even after the anesthetic stage in rats. Arq Bras Med Vet Zootec.

[32] Wang T, Zheng Q, Yang Q, Guo F, Cui H, Hu M (2024). The metabolic clock of ketamine abuse in rats by a machine learning model. Sci Rep.

[33] Veilleux-Lemieux D, Castel A, Carrier D, Beaudry F, Vachon P (2013). Pharmacokinetics of ketamine and xylazine in young and old Sprague-Dawley rats. J Am Assoc Lab Anim.

[34] Kazdağli H, Özel HF, Özbek M, Alpay Ş, Alenbey M (2022). Classical heart rate variability and non-linear heart rate analysis in mice under napentobarbital and ketamine/xylazine anesthesia. Turk J Med Sci.

[35] Barboza ARA (2022). Análise da variabilidade da frequência cardíaca e pressão arterial, em ratos, durante a anestesia por ketamina e xilazina, uretana ou isoflurano [Dissertation of Masters in Physiology, University of São Paulo].

[36] Andropoulos DB, Mossad EB, Andropoulos DB, Stayer S, Mossad EB, Miller‐Hance WC (Orgs.) (2015). Anesthesia for Congenital Heart Disease.

[37] Rodrigues SF, de Oliveira MA, Martins JO, Sannomiya P, Tostes RC, Nigro D (2006). Differential effects of chloral hydrate- and ketamine/xylazine-induced anesthesia by the s.c. route. Life Sci.

[38] Shahroozian E, Davoudi M, Hayati F, Asghar S (2017). Effects of short-term repeated fasting on anesthesia: an experimental animal study. Comp Clin Path.

